# Synergistic effect of Mn substitution and ball milling on NaCu_0.2_Fe_0.8−*x*_Mn_*x*_O_2_ cathode materials for sodium-ion batteries

**DOI:** 10.1039/d6ra02392d

**Published:** 2026-07-02

**Authors:** Ichrak Ben Slima, Kawthar Trabelsi, Lahcen Fkhar, Karim Karoui, Frédéric Boschini, Abdallah Ben Rhaiem, Abdelfattah Mahmoud

**Affiliations:** a Laboratory LaSCOM, University of Sfax BP1171 Sfax 3000 Tunisia abdallahrhaiem@yahoo.fr; b GREENMAT, CESAM, Institute of Chemistry B6, University of Liège 4000 Liège Belgium abdelfattah.mahmoud@uliege.be; c GREMAN UMR 7347-CNRS, IUT of Blois, University of Tours Blois France

## Abstract

This work focuses on the systematic investigation of the impact of Mn substitution and ball milling on NaCu_0.2_Fe_0.8−*x*_Mn_*x*_O_2_ (*x* = 0.4, 0.5, 0.6, and 0.7) cathodes. The materials were synthesized using the conventional solid-state method. The particle size was subsequently reduced through a ball-milling process. Structural characterization revealed that the samples crystallized in a hexagonal system (space group *R*3̄*m*). O3-type and P3-type layered structures were identified for samples with *x* = 0.4 and 0.5 and for samples with *x* = 0.6 and 0.7, respectively. After the ball milling process, the samples with *x* = 0.4 and 0.5 retained their O3-type structure, whereas those with *x* = 0.6 and 0.7 transformed into O3 and P2 structure types, respectively. The evolution of particle size was monitored using laser granulometry during ball milling, and morphological studies corroborated the effectiveness of ball milling in terms of breaking down of particle agglomerates. Electrochemical analyses demonstrated an enhancement in the specific capacity of NaCu_0.2_Fe_0.8−*x*_Mn_*x*_O_2_ (*x* = 0.4, 0.5, 0.6, and 0.7) materials with increasing Mn content (the sample with *x* = 0.7 delivered a capacity of 100 mAh g^−1^ at a C/20 rate and retained about 88% of its capacity after 100 cycles). Additionally, analysis of the influence of the particle size achieved through the ball milling process evidenced the effectiveness of this process in promoting the specific capacity, especially at high C-rates. Moreover, potentiostatic electrochemical impedance spectroscopy measurements proved the contributions of the electrode/electrolyte interface, charge-transfer resistance, and sodium-ion diffusion to performance. Finally, the SEM micrographs of NaCu_0.2_Fe_0.8−*x*_Mn_*x*_O_2_ (*x* = 0.7) did not show significant morphological changes in the electrode before and after cycling, confirming the high structural stability of our prepared electrodes during cycling.

## Abstract 2

This work focuses on the systematic investigation of the impact of Mn substitution and ball milling on NaCu_0.2_Fe_0.8−*x*_Mn_*x*_O_2_ (*x* = 0.4, 0.5, 0.6, and 0.7) cathodes for Na-ion batteries.

## Graphical abstract

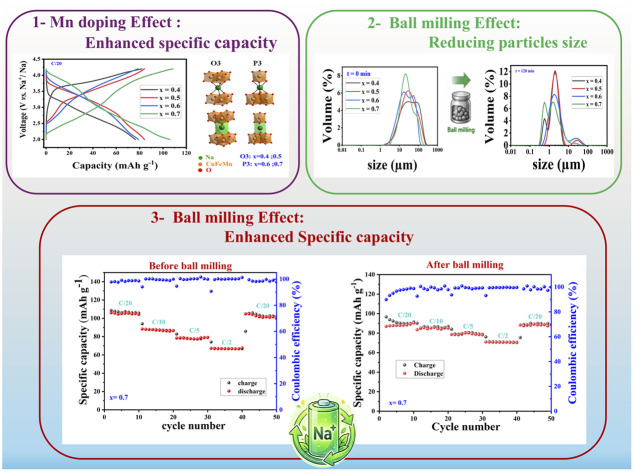

## 1 Introduction

With the booming growth of portable devices and the evolving landscape of electric vehicles, batteries are becoming a growing need. In this context, the most well-known and widely used battery technology is lithium-ion batteries. Indeed, they are highly successful and have a strong commercial presence.^1^ This is basically due to their excellent electrochemical performance, great safety and long-life cycle.^2–4^ However, this technology suffers from certain limitations, such as limited lithium sources in the Earth's crust and its high price, which inhibit its continuous production.^5^ Therefore, researchers are focused on searching for alternatives that fit the following criteria: unlimited sources, environmental friendliness, good cycling stability and low costs.^6–8^ In this context, sodium-ion batteries meet these requirements because the properties of sodium are similar to those of lithium.^9–11^ Furthermore, on the one hand, for battery commercialization, the positive electrode material represents a challenge since it is the most expensive component of a battery and accounts for almost 32% of the total cost, twice that of the anode.^12^ Accordingly, the positive electrode material must satisfy several requirements, as reported by Liu *et al.*^13^ On the other hand, the diffusion barrier of Na^+^ in layered structures may be lower than that of its Li^+^ counterpart, according to a DFT calculation used to examine the variations in the voltage, phase stability, and diffusion barriers of sodium-ion- and lithium-ion-based batteries.^14^ This result indicates that layered oxide materials, which are supported by several researchers, have a huge potential as sodium-ion battery cathodes.^13–17^ Additionally, these materials are endowed with various properties owing to the existence of different transition metal elements in their chemical structure. With the aim of taking the optimal advantage of their benefits, studies have centered around layered Na_*x*_MO_2_ with two, three or multiple transition metals in an attempt to discover suitable candidates with an increased capacity, a long cycle life, and structural stability.^15^

Accordingly, sodium sheets and MO_2_ sheets, including MO_6_ octahedra, alternate to form layered Na_*x*_MO_2_. Relying on the sodium ion's environment and oxygen's stacking sequence number, typical Na_*x*_MO_2_ is categorized into O2/O3 and P2/P3 structure types, where ‘O’ and ‘P’ imply that sodium ions occupy octahedral and prismatic sites, respectively.^18^ Moreover, certain elements that are employed as the insertion host in the production of Li layered oxides are inactive. To achieve sodium intercalation, however, their Na counterparts may need to combine many 3d transition-metal (TM) elements (Cu, Ti, V, *etc.*), and these elements exhibit active electrochemical performance.^19^

For instance, the operating voltage of NaCoO_2_ proved to be lower than the operating voltage of LiCoO_2_. Additionally, LiFeO_2_ is electrochemically inactive in a lithium cell, while NaFeO_2_ is electrochemically active and delivers a reversible capacity of about 80 mAh g^−1^ within the voltage window between 2 V and 4 V.^17,20^ Furthermore, owing to the high potential of Ni^2+^/Ni^3+^ and Ni^3+^/Ni^4+^ redox couples, both LiNiO_2_ and NaNiO_2_ display a high energy density as well as a high reversible capacity. However, the performance of LiNiO_2_ is reduced by the mixture of Li and Ni. Similarly, NaNiO_2_ exhibits low coulombic efficiency and multiple irreversible and reversible phase transitions.^21^ Likewise, huge differences are inferred in the electrochemical behavior of LiMnO_2_ and NaMnO_2_. LiMnO_2_ suffers from fast capacity decay and irreversible phase transitions, whereas NaMnO_2_ exhibits better cycling stability and a higher reversible capacity.^22^ Additionally, in LiCuO_2_, the extraction/insertion of Li is irreversible due to the formation of CuO and oxygen, which are very stable and do not react with Li reversibly. However, NaCuO_2_, with a very fast capacity decay, can reversibly extract about 0.6 mol of Na ions. It is noteworthy that copper is mixed with other metals to enhance the structural stability of the cathode material.^23–25^

Even though the single-metal Na_*x*_MO_2_ sample demonstrates pertinent results, there are still several features that need to be understood. To the best of our knowledge, mixing metals is among the best strategies to enhance the electrochemical performance of layered oxides based cathode materials for Na-ion batteries. For instance, combining Mn and Ni generates a broad voltage window by relying on the Ni^2+^/Ni^3+^/Ni^4+^ redox process, while the inactive Mn^4+^ preserves structural integrity.^1^ Despite these improvements, samples containing both Ni and Mn still exhibit a certain structural instability.^26^ Moreover, combining Fe and Ni achieves improved cycle stability.^27^ Likewise, from cost and environmental points of view, cobalt substitution is not appropriate even though it provides excellent performance.^21,28^ However, the mixture of Fe and Mn affords improved thermodynamic stability and electrochemical performance. In fact, O3-type Na_*x*_Fe_*y*_Mn_1−*y*_O_2_ delivers a lower capacity but exhibits a longer cycle life, while P2-type Na_*x*_Fe_*y*_Mn_1−*y*_O_2_ delivers a high capacity but exhibits limited cycle stability (65% capacity retention after 50 cycles).^29^ It has been reported that while the earth-abundant elements Fe and Mn are included in P2-Na_2/3_Fe_1/3_Mn_2/3_O_2_, the latter struggles to reach its maximum theoretical potential, and its stability is still not excellent. According to earlier studies, cobalt may stabilize cycling performance but reduce specific capacity, while nickel can increase specific capacity at the expense of stability. Achieving a balance between both is crucial because other elements, like Ti, Cu, Zr, and Zn, serve to increase either the specific capacity or stability.^19^

From this perspective, with regard to their natural abundance, their environmental friendliness, the high working voltage of Fe^3+^/Fe^4+^, and the high specific capacity of Mn^3+^/Mn^4+^, Fe-/Mn-based layered oxides are appealing systems from a practical viewpoint.^30^ Despite these benefits, these compounds present numerous issues, including hygroscopic characters, structural distortion due to the Jahn–Teller effect and irreversible phase transitions.^31–33^ Diverse approaches have been examined in order to address these issues. For instance, Luo *et al.* demonstrated that structure-kinetics co-optimization is possible through the careful regulation of sodium's stoichiometry. Accordingly, they investigated the cathode material Na_*x*_Ni_1/3_Fe_1/3_Mn_1/3_O_2_ (*x* = 0.67, 0.8, 0.9, 1.0). When the Na_f_/Na_e_ ratio of the Na_0.9_Ni_1/3_Fe_1/3_Mn_1/3_O_2_ material reaches 0.55, the electrochemical performance is at its peak, and the Na_f_/Na_e_ ratio decreases. Following the multiplicative performance test, its discharge specific capacity decreases to 95.7 mA g^−1^, from its original discharge specific capacity of 112 mA g^−1^.^34^ Moreover, it is worth noting that the method of synthesis impacts electrochemical characteristics. Indeed, solid-state and spray-drying methods have been used to create P2-Na_0.67_Mn_0.5_Fe_0.3_Mg_0.2_O_2_. In contrast to traditional solid-state synthesis, spray-drying seems to be quite promising for producing materials with morphologies and microstructures more suited for use as battery cathodes. For P2-Na_0.67_Mn_0.5_Fe_0.3_Mg_0.2_O_2_ synthesized by spray-drying, the Na diffusion coefficient (*D*_Na^+^_) is more than two orders of magnitude greater than that of the solid-state synthesized material (10^−8^*vs.* 10^−10^ cm^2^ s^−1^).^35^ Additionally, electrochemical characteristics may be affected by the synthesis process. For example, P2-Na_2/3_Fe_1/2_Mn_1/2_O_2_'s quick cooling process effectively prevents the formation of Mn vacancies, which has a major effect on decreasing Na layers. The NFM-Q sample's excellent ion transport kinetics are further validated by its greater sodium ion diffusion coefficient and reduced charge transfer impedance. Significant multiplicity performance is corroborated by the NFM-Q sample, which exhibits an impressive discharge capacity (192.7 mAh g^−1^), cycling stability (123.8 mAh g^−1^, 65%, after 50 cycles), energy density (574.0 Wh kg^−1^), and air stability (185.8 mAh g^−1^, 61.8%, after soaring and 50 cycles).^36^

It is also equally interesting to invest in the coating approach. In addition to limiting the dissolution of Mn and Fe ions, the coating stops H_2_O molecules and CO_2_ in the air from reacting negatively with the cathode material. Thus, after 30 days of storage in a humid environment with a relative humidity of 80%, P2-Na_0.67_Ni_0.1_Mn_0.8_Fe_0.1_O_2_@Na_0.61_MnO_2_ still exhibits a notable capacity retention rate of 55%, confirming that the coating method can shield layered oxide materials from CO_2_ and H_2_O in the air.^37^ Thus, metal doping is still the most preferred technique for dealing with layered oxide problems. In fact, the P2/O3 biphasic Fe/Mn-layered cathode material with Mg and Nb co-doping, Na_0.67_Fe_0.5_Mn_0.38_Mg_0.1_Nb_0.02_O_2_, demonstrates exceptional air stability and sodium storage characteristics. They also provide a better insight into the underlying mechanism of action. In particular, the dual function of Mg and Nb co-doping includes limiting Na^+^/H^+^ exchange and improving sodium ion kinetics. In addition to its remarkable capacity (162.3 mAh g^−1^, 0.1C), stability (87.8%, 100 cycles), rate (84.2%), and energy density (325.7 Wh kg^−1^), Na_0.67_Fe_0.5_Mn_0.38_Mg_0.1_Nb_0.02_O_2_ exhibits superior air stability (114.1 mAh g^−1^, 84.6%, RH60%-1 week).^38^ Additionally, Mg doping has the potential to regulate the Na_e_/Na_f_ ratio, maintain an intact P2 phase of the Na_0.67_(Ni_0.1_Mn_0.8_Fe_0.1_)_1−*x*_Mg_*x*_O_2_ (*x* = 0.04) structure during a high-voltage charging process, and exhibit a remarkable initial capacitance (119.5 mAh g^−1^, 0.1C), stability (80.0% over 200 cycles), and energy density (356.5 Wh kg^−1^).^39^ Ti-ion doping may also greatly improve the structural stability of the Fe-/Mn-based cathodes, inhibit the production of Fe^4+^O_6_ in the sodium-ion layer, and promote Na^+^ migration. The Na_0.67_(Fe_0.5_Mn_0.5_)_0.495_Ti_0.05_O_2_ cathode exhibits a discharge capacity of 182.7 mAh g^−1^ and a reduced cell volume change (1.26%) during cycling.^40^ Moreover, copper substitution is well sought for as it is inexpensive, harmless, and has a wide range of potential applications.^41^ Indeed, integrating the Cu^2+^/Cu^3+^ redox couple in P2-Na_7/9_(Mn_2/3_Fe_1/9_Cu_2/9_)O_2_ and O3-Na_0.9_Cu_0.22_Fe_0.3_Mn_0.48_O_2_ reinforces the effectiveness of inhibiting oxidation by CO_2_, O_2_, and H_2_O to promote the average stored voltage, prevent spontaneous O3–P3 phase transitions and boost cycling performance.^42,43^ Experimentally, P2-Na_7/9_(Mn_2/3_Fe_1/9_Cu_2/9_)O_2_ exhibits a voltage range of 2.5–4.2 V and, at a 0.1C rate, a reversible capacity of 89 mAh g^−1^ and a capacity retention of 85% after 150 cycles. O3-Na_0.9_Cu_0.22_Fe_0.3_Mn_0.48_O_2_ delivers a voltage range of 2.5–4.05 V and, at a 0.1C rate, a reversible capacity of 100 mAh g^−1^ and a capacity retention of 97% after 100 cycles.^44,45^

As far as the current research paper is concerned, the first section is devoted to examining NaCu_0.2_Fe_0.8−*x*_Mn_*x*_O_2_ samples as cathode active materials for sodium-ion batteries. It explores the effect of Mn substitution on the structural and electrochemical properties of these compounds. Moreover, it is commonly recognized that the shape and size of the electrode materials' particles have substantial effects on their electrochemical qualities.^46^ From this perspective, to reduce the particle size and promote Na^+^ ion transport in the electrode during the charge/discharge process, the generated materials were subjected to a ball-milling process. Section two, therefore, centres around how these materials' particle sizes affect their electrochemical capabilities.

This investigation was carried out by combining different characterization techniques such as XRD, SEM, laser granulometry, BET analysis, and Mössbauer spectroscopy in order to assess the samples' structure, morphology, particle size, specific surface area, iron valence state and chemical environment. To examine the electrochemical properties of NaCu_0.2_Fe_0.8−*x*_Mn_*x*_O_2_ materials, galvanostatic tests were performed in a half-cell configuration against the sodium metal, and cyclic voltammetry was performed on the prepared samples.

## 2 Experimental section

### 2.1 Materials synthesis

NaCu_0.2_Fe_0.8−*x*_Mn_*x*_O_2_ was synthesized using a solid-state method: a mixture of Na_2_CO_3_ (Sigma-Aldrich, 99%), CuO (Sigma-Aldrich, 99%), Fe_2_O_3_ (Sigma-Aldrich, 99%) and Mn_2_O_3_ (Sigma-Aldrich, 99%) was prepared in a proportional ratio and heated in air at 850 °C (more details are provided in our previous work^47^).

### 2.2 Structural and morphological characterization

A Bruker D8 Discover Twin–Twin was used at room temperature with Cu K_α_ radiation (*λ* = 1.5406 Å, 10° ≤ 2*θ* ≤ 80°) in order to check the purity of the prepared samples. A scanning electron microscope (XL30 FEG ESEM, FEI) was employed at an accelerating voltage of 15 kV under high vacuum to investigate the samples' morphology. A Malvern Mastersizer was used for granulometry measurements. The powders were mixed with isopropanol and sonicated for 1 minute. Every sample was analyzed three times, separated by 10 seconds. Thus, the findings reported in this paper correspond to an average of those three assessments. The surface area of the prepared materials was measured using a surface area analyzer (Micrometrics, ASAP 2020). Before the measurement, the powders were degassed at 150 °C for 12 h under a nitrogen flow to remove all the moisture that may be adsorbed on the surface of the materials. The mono-point method was used in the current research work in order to determine the specific surface area.

Iron-57 Mössbauer spectra were obtained using a constant-acceleration spectrometer with a ^57^Co (Rh) source at room temperature. The Mössbauer spectral absorbers were prepared with ∼40 mg cm^−2^ of the NaCu_0.2_Fe_0.8−*x*_Mn_*x*_O_2_ material mixed with boron nitride. The spectrometer was calibrated at room temperature using an α-iron foil. The measurements were carried out within the velocity range of ±4 mm s^−1^ with an optimal energy resolution at room temperature. The room-temperature Mössbauer spectra were fitted with three Lorentzian doublets using the Fullham program. The validity of fits was specified by relying on minimizing the number of parameters and *χ*^2^ values (*χ*^2^ < 1).

### 2.3 Electrochemical characterization

The positive electrodes were prepared by mixing 70 wt% of the NaCu_0.2_Fe_0.8−*x*_Mn_*x*_O_2_ active material, 20 wt% of the polyvinylidene fluoride (PVDF, Aldrich, 99.99%) binder and 10 wt% of Super-P carbon with an appropriate amount of *N*-methyl-2-pyrrolidone (NMP, purity = 99.9%). The mixture was vigorously mixed in a Speed Mixer machine. The obtained black viscous slurry was coated onto an aluminum foil (thickness: ∼120 µm), followed by drying under vacuum at 110 °C for 12 h. The dry laminate electrodes were then punched into disks with a 12.6 mm diameter (active mass of approximately 3 mg). The electrochemical analyses of the obtained electrodes were performed in CR2032 coin-type half-cells assembled with sodium chips (diameter = 12 mm) as counter electrodes and a 1.6 µm monolayer Whatman GF/A separator in an Ar-filled glove box (H_2_O < 0.1 ppm, O_2_ < 0.1 ppm). The electrolyte considered in this study was NaPF_6_ in propylene carbonate (PC) with 5 wt% fluoroethylene carbonate (FEC). Galvanostatic charge/discharge characterizations were performed at room temperature using a multichannel BTS4000 Neware electrochemical test system in the fixed voltage window of 2–4.2 V *vs.* Na^+^/Na (theoretical capacity (*C*_theo_) = 240 mAh g^−1^ was considered). Cyclic voltammetry (CV) tests were conducted at room temperature using a BioLogic battery cycler within a test voltage range of 2–4.2 V at different scan rates between 0.1 and 5 mV s^−1^. Potentiostatic electrochemical impedance spectroscopy (PEIS) measurements were carried out at room temperature using a BioLogic VMP3 potentiostat with a 5 mV amplitude in the frequency range from 100 kHz to 100 MHz in a three-electrode Swagelok cell. Aftermath software was used for equivalent circuit modeling.

## 3 Results and discussion

### 3.1 Structural and electrochemical properties of NaCu_0.2_Fe_0.8−*x*_Mn_*x*_O_2_ samples (*x* = 0.4, 0.5, 0.6, and 0.7)

**Fig. 1:**
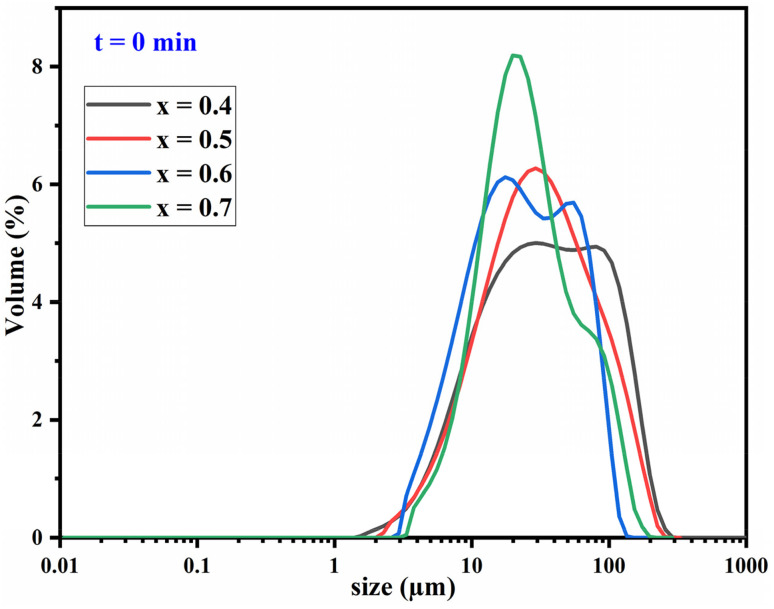
Particle size distributions of the NaCu_0.2_Fe_0.8−*x*_Mn_*x*_O_2_ samples (*x* = 0.4, 0.5, 0.6, and 0.7).

The X-ray diffraction patterns of NaCu_0.2_Fe_0.8−*x*_Mn_*x*_O_2_ samples (*x* = 0.4, 0.5, 0.6, 0.7) recorded at room temperature are depicted in Fig. S1.^47^ These compounds crystallize in the hexagonal system in the *R*3̄*m* space group. A variation in the cell parameters (Table S1) induces a decrease in the volume of the sodium site with increasing Mn amount in the samples.^47^ Furthermore, the analysis of the crystalline structures of these compounds shows that the samples with *x* = 0.4 and 0.5 are of the O3 structure type, in which sodium ions occupy octahedral sites and NaO_6_ octahedra share edges, whereas the samples with *x* = 0.6 and 0.7 are of the P3 structure type, in which sodium ions occupy prismatic sites and NaO_6_ prisms share edges and faces.^47^ Additionally, using scanning electron microscopy, the morphology of the prepared samples was examined. Fig. S2 shows the SEM micrographs of NaCu_0.2_Fe_0.8−*x*_Mn_*x*_O_2_ samples (*x* = 0.4, 0.5, 0.6, 0.7) recorded at room temperature. As can be seen from the SEM images, particles with an irregular shape are observed in all samples, and only one contrast is detected, indicating similar chemical compositions. The remarkable agglomeration of the primary particles in all compounds is equally observed. The particle size and particle size distributions of NaCu_0.2_Fe_0.8−*x*_Mn_*x*_O_2_ compounds were determined using laser granulometry. The obtained results are outlined in Fig. 1, where the particle distribution is agglomerated in the range of 20–35 µm (*d*_50_). For the samples with *x* = 0.4, 0.6, and 0.7, there is a bimodal distribution, indicating the existence of two populations of particles with different concentrations. The sample with *x* = 0.5 exhibits a Gaussian particle size distribution.

According to the granulometry parameters displayed in Table 1, the *d*_50_ value decreases from 33.5 µm to 22.9 µm as the Mn content increases from *x* = 0.4 to *x* = 0.6. A slight increase to 23.9 µm is observed at *x* = 0.7; however, this variation remains relatively small and is not statistically significant. In contrast, the *d*_90_ value shows a more noticeable increase at *x* = 0.7, indicating the presence of larger particles or agglomerates. Overall, these results suggest a general reduction in the particle size with increasing Mn content, accompanied by agglomeration effects at higher substitution levels.

**Table 1:** *d*_10_, *d*_50_, and *d*_90_ parameters of the NaCu_0.2_Fe_0.8−*x*_Mn_*x*_O_2_ materials obtained from granulometry laser measurements

Sample	*d*_10_ (µm)	*d*_50_ (µm)	*d*_90_ (µm)
*x* = 0.4	8.36	33.50	119.00
*x* = 0.5	8.73	30.00	100.00
*x* = 0.6	7.19	22.90	68.90
*x* = 0.7	9.55	23.90	78.40

**Fig. 2:**
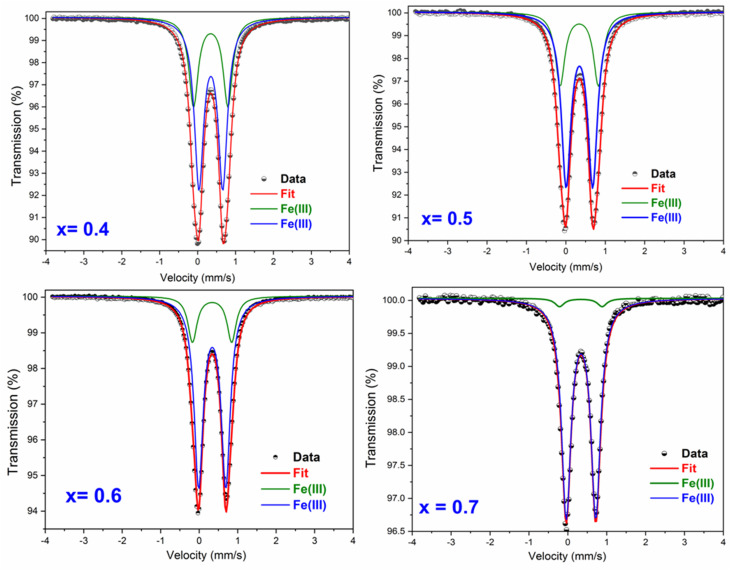
Mössbauer spectra of the NaCu_0.2_Fe_0.8−*x*_Mn_*x*_O_2_ samples (*x* = 0.4, 0.5, 0.6, and 0.7) recorded at room temperature.

The study of the iron valence state and its local environment is essential, as is the study of the structure and morphology of the samples. For this reason, the Mössbauer spectra of NaCu_0.2_Fe_0.8−*x*_Mn_*x*_O_2_ compounds (*x* = 0.4, 0.5, 0.6, 0.7) recorded at room temperature are plotted in Fig. 2. The Mössbauer spectra of all samples consist of an asymmetric doublet. The hyperfine parameters are summarized in Table 2. The isomer shift (*δ*) and quadrupole splitting (*Δ*) values are indicative of high-spin Fe^3+^ in octahedral ligand fields located at two distinct sites, designated as Fe^3+^(1) and Fe^3+^(2).^48,49^ A comparison of the quadrupole splitting between Fe^3+^(1) and Fe^3+^(2) reveals that the Fe^3+^(1) site has a larger value of quadrupole splitting, which suggests that the disorder in the environment has a more significant impact on the local environment of the FeO_6_ octahedron than on the Fe^3+^(2) site. Additionally, this finding demonstrates that the valence state of Fe is not affected by the substitution of Mn and that the nominal valence state for all the compounds is NaCu_0.2_^2+^[Fe_*y*_^3+^(1)Fe_1−*y*_^3+^(2)]_0.8−*x*_Mn_*x*_^3+^O_2_. Indeed, as the Mn percentage increases, the quadrupole splitting of both Fe sites increases. Consequently, when the Mn amount rises, the density of the 3d electrons of Fe decreases even when the valence state of Fe is maintained.^50,51^

**Table 2:** Hyperfine parameters of the room-temperature Mössbauer spectra of the NaCu_0.2_Fe_0.8−*x*_Mn_*x*_O_2_ samples (*x* = 0.4, 0.5, 0.6, and 0.7)

Sample	Species	*δ* (mm s^−1^)	*Δ* (mm s^−1^)	*Γ* (mm s^−1^)	Area (%)
*x* = 0.4	Fe(iii)_1_	0.34 (1)	0.90 (1)	0.29 (1)	35 (1)
	Fe(iii)_2_	0.35 (2)	0.62 (1)	0.29 (1)	65 (1)
*x* = 0.5	Fe(iii)_1_	0.34 (1)	0.97 (1)	0.29 (1)	30 (1)
	Fe(iii)_2_	0.34 (2)	0.67 (1)	0.29 (1)	70 (1)
*x* = 0.6	Fe(iii)_1_	0.33 (1)	1.01 (2)	0.28 (1)	20 (1)
	Fe(iii)_2_	0.34 (2)	0.71 (1)	0.28 (1)	80 (1)
*x* = 0.7	Fe(iii)_1_	0.34 (1)	1.10 (2)	0.29 (1)	4 (1)
	Fe(iii)_2_	0.34 (2)	0.76 (1)	0.29 (1)	96 (1)

**Fig. 3:**
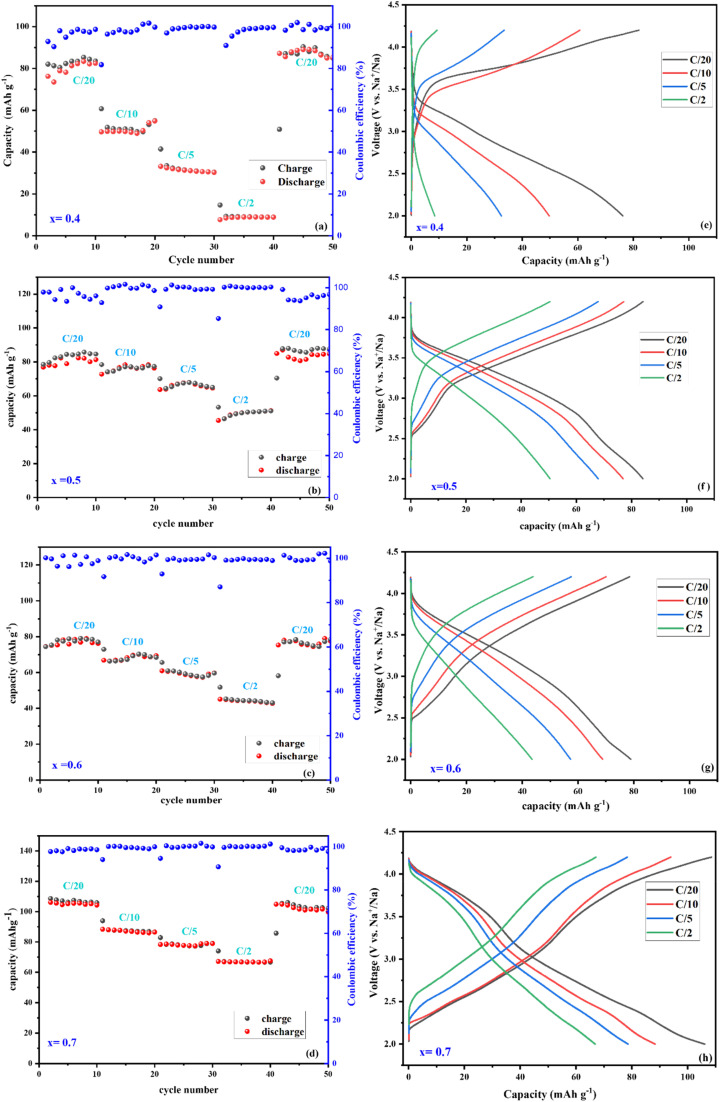
(a–d) Rate capability and (e–h) voltage profiles of the NaCu_0.2_Fe_0.8−*x*_Mn_*x*_O_2_ samples (*x* = 0.4, 0.5, 0.6, and 0.7).

The electrochemical performance of NaCu_0.2_Fe_0.8−*x*_Mn_*x*_O_2_ materials was assessed by galvanostatic charge–discharge cycling in a half-cell configuration within the voltage range of 2–4.2 V *vs.* Na^+^/Na. The rate capability of NaCu_0.2_Fe_0.8−*x*_Mn_*x*_O_2_ electrodes was assessed at various rates from C/20 to C/2. Fig. 3 and Table 3 illustrate the delivered reversible discharge capacities. As the C-rate increases, the reversible discharge capacity gradually declines, and the compound with *x* = 0.4 exhibits the lowest capacity. At the C/20 rate, the sample with *x* = 0.4 delivers a discharge capacity of 76 mAh g^−1^, whereas the sample with *x* = 0.7 delivers a capacity of 106 mAh g^−1^. At the C/2 rate, they deliver 8 mAh g^−1^ and 66 mAh g^−1^, respectively. At this stage, the effect of the crystalline structure type appears, where the P structure (*x* = 0.7) facilitates the migration path of Na^+^ ions, owing to the direct movement from one site to another, which is unlike the O structure type, where Na^+^ ions make a zigzag path as they move from one site to another. When the current density rate returns to C/20, the capacities return to their initial values, which proves the good structural stability of our materials during electrochemical cycling at high C-rates.

**Table 3:** Discharge specific capacity obtained at various C-rates for the NaCu_0.2_Fe_0.8−*x*_Mn_*x*_O_2_ samples (*x* = 0.4, 0.5, 0.6, and 0.7)

Sample	Specific capacity (mAh g^−1^)				(C/2)/(C/20) (%)
	C/20	C/10	C/5	C/2	
*x* = 0.4	75.96	49.60	32.56	8.24	10.48%
*x* = 0.5	84.24	76.78	67.77	50.34	59.75%
*x* = 0.6	78.76	68.93	53.30	43.73	55.52%
*x* = 0.7	106	88.28	78.55	66.37	62.61%

Furthermore, as the C-rate increases, the voltage profiles (Fig. 3e–h) start at a higher voltage, which can be attributed to the time of insertion and deinsertion of sodium ions, which increases, thereby occurring at high voltages. However, as the Mn amount increases (*x* = 0.7), the voltage profiles start at a lower voltage (Fig. 3), which is due to the structural change in sodium sites, which become prismatic sites having a lower volume of Na-sites and generating a faster ionic and electronic conductivity.^47^ Besides, the sample with *x* = 0.7 has a higher Mn content. This refers to the differences in the electrochemical activity of Fe and Mn and the low working voltage of Mn, which will provide a large part of the capacity as it starts at a low voltage.^19^ Additionally, the decrease in the specific discharge capacity with increasing C-rates reveals that the ohmic resistance becomes considerable, inducing the increase in the polarization of the electrode, which can be observed in the voltage profiles as the change in the curves' slopes.^52,53^

The rate capability features indicate that the specific capacity increases with an increase in the Mn percentage. This finding is quite intriguing with respect to the cycling performance of the sample with *x* = 0.7, which delivers the highest capacities at different C-rates. Fig. S3 shows the evolution of the specific capacity with the cycle number for the NaCu_0.2_Fe_0.8−*x*_Mn_*x*_O_2_ sample (*x* = 0.7) at the C/20 rate and room temperature. The delivered initial charge and discharge capacities are 106 and 100 mAh g^−1^, respectively. After 100 cycles, this sample maintains about 88% of its initial capacity, which reflects, in addition to its higher coulombic efficiency (>98%), the structural reversibility during cycling of the sample with *x* = 0.7.

**Fig. 4:**
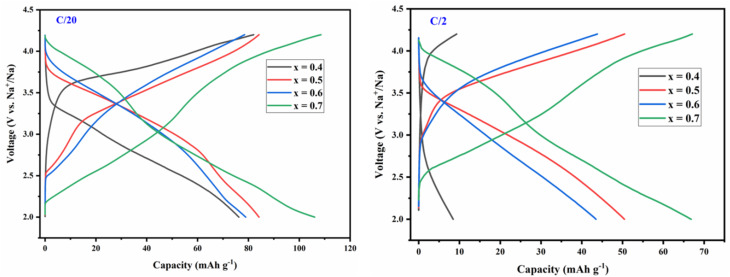
Voltage profiles of the NaCu_0.2_Fe_0.8−*x*_Mn_*x*_O_2_ samples (*x* = 0.4, 0.5, 0.6, and 0.7) at C/20 and C/2 rates.

In addition, as the Mn percentage increases (Fig. 4), the discharge capacity increases, resulting in an increase in the capacity retention from 10.4% for the sample with *x* = 0.4 to 63% for the sample with *x* = 0.7 (Table 3). This can be attributed to the influence of the Mn amount on the structure of these samples, which switches from the O3 type for samples with *x* = 0.4 and 0.5 to the P3 type for samples with *x* = 0.6 and 0.7, leading to a change in the site of sodium from octahedral to prismatic, which accordingly enhances Na^+^ ion diffusion and facilitates their migration paths. These results are corroborated by the electrochemical study, in which an enhancement in the electrical conductivity with increasing Mn percentage is notably observed.^47^ Likewise, a little drop in the reversible discharge capacity is detected when the Mn percentage rises from *x* = 0.5 to *x* = 0.6. This might be due to the particle size distribution presented in Fig. 1, which is more homogenous in the *x* = 0.5 sample compared to the *x* = 0.6 sample.

To recapitulate, Mn substitution in NaCu_0.2_Fe_0.8−*x*_Mn_*x*_O_2_ (*x* = 0.4, 0.5, 0.6, 0.7) materials enhances the specific capacity and capacity retention at both low (C/20) and high rates (C/2). At a rate of C/10, P3-NaCu_0.2_Fe_0.1_Mn_0.7_O_2_ (*x* = 0.7) delivers a specific capacity of 88 mAh g^−1^ within the voltage range of 2–4.2 V. This is a little higher than that previously reported for P2/O3-Na_0.78_Ni_0.2_Fe_0.38_Mn_0.42_O_2_ and P3/O3-Na_2/3_Ni_1/3_Mn_1/3_Ti_1/3_O_2_, both of which deliver a specific capacity of 86 mAh g^−1^ within the voltage ranges of 2.5–4 V and 2.5–4.15 V, respectively.^54^ In order to gain a deeper insight into the redox reactions of the transition metal cation in the first cycle, d*Q*/d*V* curves of the samples with *x* = 0.4 and *x* = 0.7 are plotted in Fig. S4. The redox reactions can be split into four ranges, and the redox peaks are made up of the redox couples Mn^3+^/Mn^4+^ (green color) and Fe^3+^/Fe^4+^(pink color). The oxidation peaks below 3 V belong to the Mn^3+/4+^ redox couple, while the reduction peaks above 2.8 V are assigned to the Fe^3+/4+^ redox couple.^55–57^ It should be noted that the absence of the copper redox pair in the d*Q*/d*V* curves indicates Cu^2+^'s lack of electrochemical activity.^58^

**Fig. 5:**
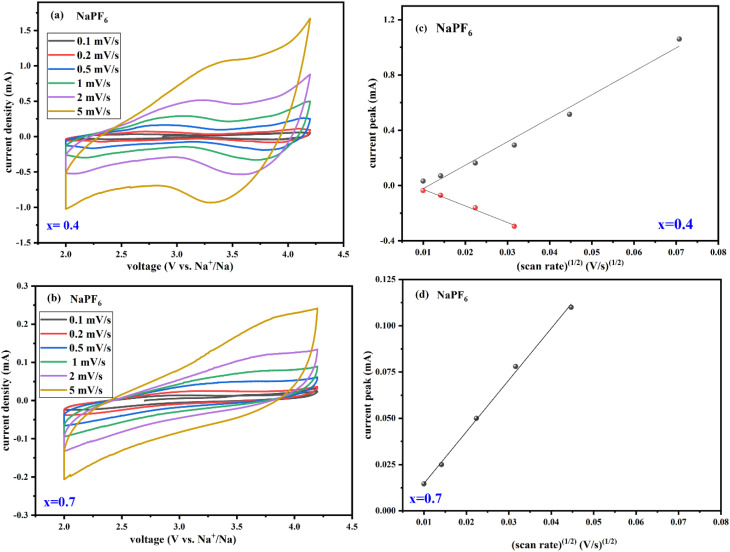
(a and b) Cyclic voltammetry at various scan rates and (c and d) linear relationship between the peak current and scan rate of the NaCu_0.2_Fe_0.8−*x*_Mn_*x*_O_2_ cathode materials for Na-ion batteries (*x* = 0.4 and 0.7).

Cyclic voltammetry (CV) is commonly used to identify the redox properties during electrochemical processes and is sensitive to phase transformation.^59^ The CV curves of the NaCu_0.2_Fe_0.8−*x*_Mn_*x*_O_2_ electrodes (*x* = 0.4 and 0.7) within the voltage range of 2–4.2 V during the first cycle were plotted at different scan rates (0.1, 0.2, 0.5, 1, 2 and 5 mV s^−1^) and are depicted in Fig. 5. As the scan rate increases, the redox peaks become steady and exhibit a little shift, indicating that NaCu_0.2_Fe_0.8−*x*_Mn_*x*_O_2_ materials have great structural stability, and only a low polarization is detected. Moreover, the peak currents of the cathodic and anodic peaks gradually increase as the scan rate rises. It is worth noting that although the redox peaks appear less pronounced in the CV curves of the *x* = 0.7 sample, the corresponding d*Q*/d*V* analysis confirms the presence of the Fe^4+^/Fe^3+^ redox couple. The reduced intensity of these peaks in the CV curves is attributed to the lower Fe content and the increased overlap with broader Mn-related electrochemical processes. The correlation between the peak current and the square root (*v*^1/2^) of the scan rate was established using linear regression analysis, which is shown in Fig. 5. As for the sodiation and desodiation processes, all the peak currents of both materials exhibit a convenient linear dependency on the square root of the scan rate (*v*^1/2^), which is suggestive of diffusion-controlled features.^60,61^

### 3.2 Influence of the ball milling process on the properties of NaCu_0.2_Fe_0.8−*x*_Mn_*x*_O_2_ samples (*x* = 0.4, 0.5, 0.6, and 0.7)

**Fig. 6:**
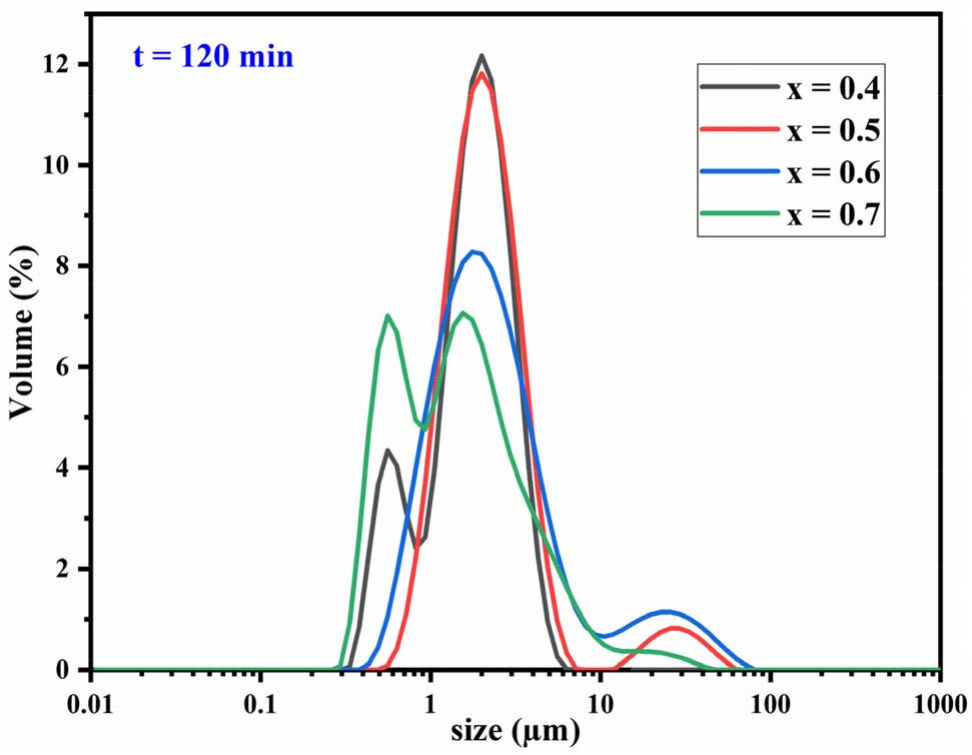
Particle size distributions of the ground NaCu_0.2_Fe_0.8−*x*_Mn_*x*_O_2_ samples (*x* = 0.4, 0.5, 0.6, and 0.7).

In the subsequent experiments, the basic objective was to enhance the electrochemical performance of NaCu_0.2_Fe_0.8−*x*_Mn_*x*_O_2_ samples by reducing the particle size, thereby increasing their surface area. For this reason, the NaCu_0.2_Fe_0.8−*x*_Mn_*x*_O_2_ compounds underwent a ball milling process for 2 h at 350 rpm (with steps of 30 min) using a planetary mill (Retsch PM400/2, alternate rotation mode). The room-temperature XRD patterns of NaCu_0.2_Fe_0.8−*x*_Mn_*x*_O_2_ samples (*x* = 0.4, 0.5, 0.6, 0.7) after the ball milling process are displayed in Fig. S5. These samples crystallize in the hexagonal system, as demonstrated by the structural refinement performed by the Rietveld method using FullProf software. The samples with *x* = 0.4, 0.5, and 0.6 retain the *R*3̄*m* space group, but the one with *x* = 0.7 has the *P*6_3_/*mmc* space group. Table S2 provides the cell parameters and fit criteria. Additionally, following the ball milling procedure, the crystal structure of the samples reveals that the samples with *x* = 0.4 and 0.5 retain their O3 structure type, whereas the sample with *x* = 0.6 transforms from the P3 to O3 type. Furthermore, the sample with *x* = 0.7 transforms from the P3 to P2 structural type. Table S3 lists the atomic positions for each sample. Fig. S6 exhibits the SEM micrographs of NaCu_0.2_Fe_0.8−*x*_Mn_*x*_O_2_ samples after the ball milling process. After grinding, the samples have a homogeneous, uniformly distributed dispersion of particles, which is inferred because agglomeration is eradicated. Fig. 6 and Table 4 show the evolution of the particle size distribution obtained by laser granulometry. There is a noticeable change in the particle size distribution towards smaller values. The bimodal distribution is present in all samples. The efficiency of the grinding is clearly observed in the *d*_50_ parameter, which decreases significantly to around 2 µm for samples with *x* = 0.4, 0.6, and 0.7. In the case of the sample with *x* = 0.5, the *d*_50_ is still higher than 10 µm. Notably, the reduction in particle size is confirmed by both SEM and laser granulometry. This can benefit the kinetics of sodium insertion/extraction into the structure, which can positively affect the capacity at high current densities. Additionally, it is crucial to assess the samples' specific surface area, as it is known that an increase in the specific surface area occurs when the particle size decreases.

**Table 4:** *d*_10_, *d*_50_, and *d*_90_ parameters of the ground NaCu_0.2_Fe_0.8−*x*_Mn_*x*_O_2_ materials obtained from granulometry laser measurements

Sample	*d*_10_ (µm)	*d*_50_ (µm)	*d*_90_ (µm)
*x* = 0.4	0.608	1.80	3.22
*x* = 0.5	1.100	12.00	4.18
*x* = 0.6	0.909	2.11	10.90
*x* = 0.7	0.496	1.40	4.96

**Fig. 7:**
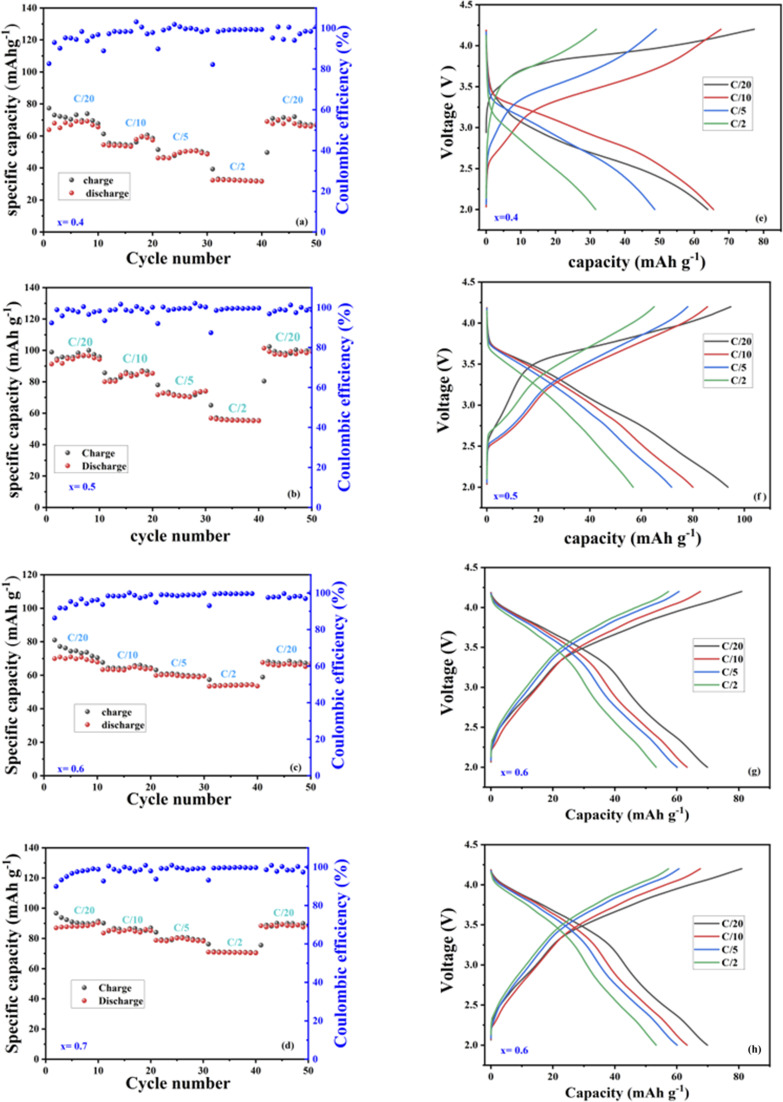
(a–d) Rate capability and (e–h) voltage profiles of the ground NaCu_0.2_Fe_0.8−*x*_Mn_*x*_O_2_ samples (*x* = 0.4, 0.5, 0.6, and 0.7).

Table 5 outlines the findings of the BET analysis that was conducted for this specific purpose. NaCu_0.2_Fe_0.8−*x*_Mn_*x*_O_2_ materials with a smaller and more uniform particle size are expected to deliver high specific capacities upon grinding. Therefore, assessing the electrochemical properties of these cathode materials was the chief target of the subsequent studies. Galvanostatic charge–discharge cycling in a half-cell configuration was used to evaluate the electrochemical performance of ground NaCu_0.2_Fe_0.8−*x*_Mn_*x*_O_2_ materials within the voltage range of 2–4.2 V *vs.* Na^+^/Na. Fig. 7 illustrates the rate performance of ground NaCu_0.2_Fe_0.8−*x*_Mn_*x*_O_2_ electrodes examined at different current densities between C/20 and C/2.

**Table 5:** Specific surface area of the ground NaCu_0.2_Fe_0.8−*x*_Mn_*x*_O_2_ materials obtained from BET analysis

Sample	*x* = 0.4	*x* = 0.5	*x* = 0.6	*x* = 0.7
Specific surface area (m^2^ g^−1^)	3.39	2.98	4.67	4.77

Table 6 summarizes the delivered reversible discharge capacities of ground NaCu_0.2_Fe_0.8−*x*_Mn_*x*_O_2_ cathode materials for Na-ion batteries. At this stage, an improvement in the specific discharge capacity is observed, particularly at rates of C/5 and C/2, compared with the electrochemical performance obtained before grinding. This leads to an increase in the capacity retention of around 82% for the sample with *x* = 0.7. Moreover, we found that the discharge capacities are larger for the sample with *x* = 0.7 because it has the highest specific area, which supports the BET analysis results. However, in the samples with *x* = 0.5 and *x* = 0.6, the specific capacity of the *x* = 0.5 sample is higher than that of the *x* = 0.6 sample, even though the *x* = 0.6 sample has the largest specific area. This might be attributed to the particle distribution, as indicated by the granulometry measurements (Fig. 6), where most particles in the *x* = 0.5 sample have the same particle size, which is confirmed by an intense peak, while the particles in the *x* = 0.6 sample show two peaks, suggesting two particle sizes.

**Table 6:** Discharge specific capacity at various C-rates for the ground NaCu_0.2_Fe_0.8−*x*_Mn_*x*_O_2_ samples (*x* = 0.4, 0.5, 0.6, and 0.7)

Sample	Specific capacity (mAh g^−1^)				C2/C20 (%)
	C/20	C/10	C/5	C/2	
*x* = 0.4	64	65.76	48.56	31.49	49.20%
*x* = 0.5	93.61	79.89	71.89	56.80	60.67%
*x* = 0.6	69.87	63.33	60.12	53.31	76.29%
*x* = 0.7	86.72	83.51	78.71	70.97	81.80%

Furthermore, at a rate of C/20, the ground NaCu_0.2_Fe_0.8−*x*_Mn_*x*_O_2_ samples maintain their reversible structural behavior, with their capacity returning to their starting values. Therefore, the ball-milling process breaks up the initially agglomerated particles, aids in their homogeneous distribution, and increases their specific surface area. It also allows the penetration of the electrolyte into the cathode, which improves the particle surface-electrolyte contact, generating more active surface materials, more active channels and more active sites at the interfaces, as well as accelerating ion diffusion pathways, thereby enhancing electrochemical properties. These findings align well with previous research studies on the impact of the particle size on a battery's electrochemical performance conducted on lithium-ion batteries using a LiNi_1/3_Mn_1/3_Co_1/3_O_2_ cathode.^62^

**Fig. 8:**
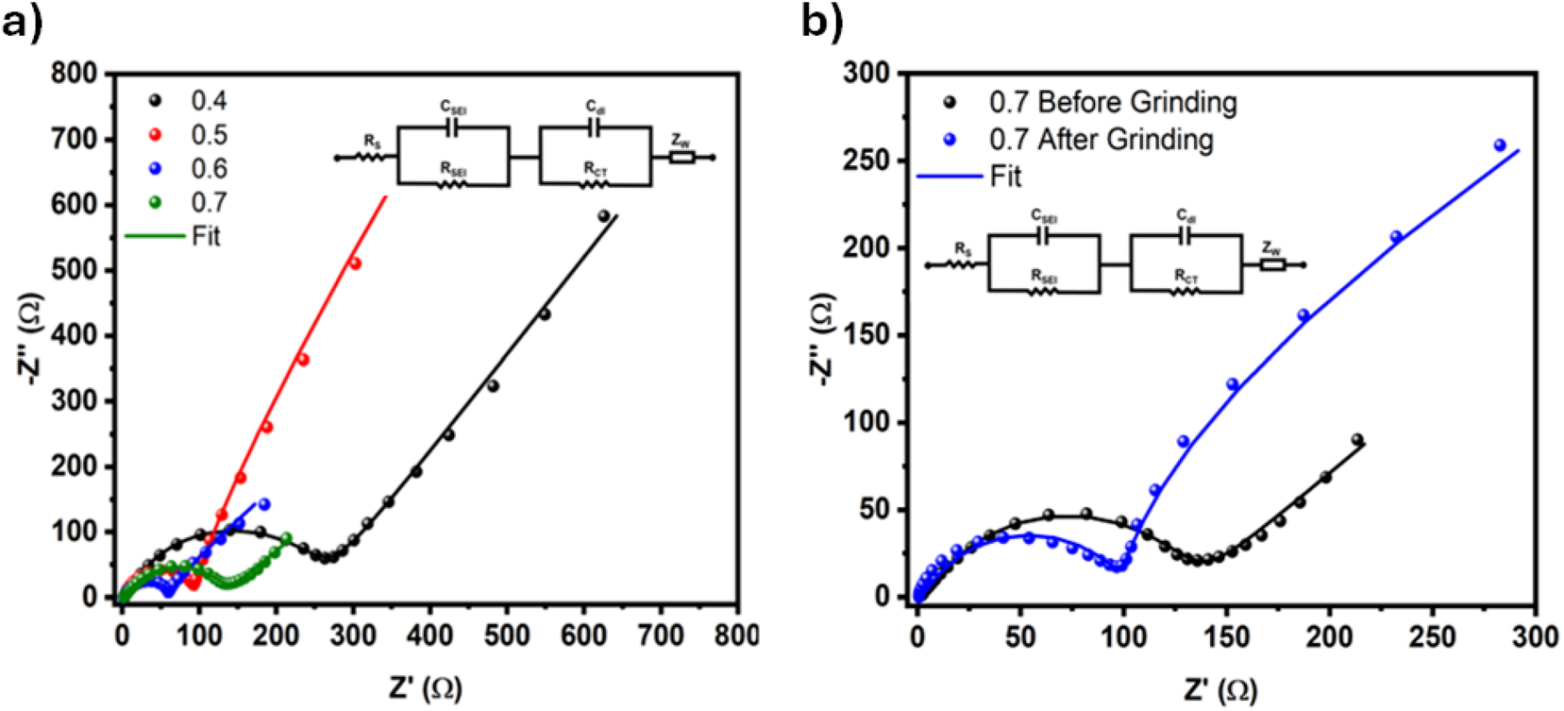
(a) Nyquist plots of the NaCu_0.2_Fe_0.8−*x*_Mn_*x*_O_2_ samples before grinding (*x* = 0.4, 0.5, 0.6 and 0.7). (b) Comparison of PEIS data of the NaCu_0.2_Fe_0.1_Mn_0.7_O_2_ (*x* = 0.7) samples before and after grinding.

Potentiostatic electrochemical impedance spectroscopy (PEIS) measurements were performed to investigate the internal resistance of the studied electrodes using a three-electrode Swagelok cell at room temperature. The results are presented in Fig. 8. The equivalent circuit (inset) was used to fit the PEIS data, and the extracted resistance values are summarized in Table 7.

**Table 7:** Fitted resistance values of the NaCu_0.2_Fe_0.8−*x*_Mn_*x*_O_2_ samples

Sample	*R*_S_ (Ω)	*R*_SEI_ (Ω)	*R*_CT_ (Ω)
*x* = 0.4	2.85	9.20	240.4
*x* = 0.5	1.74	3.57	90.1
*x* = 0.6	1.62	4.2	63.1
*x* = 0.7	1.61	7.8	120.5
*x* = 0.7 after grinding	0.9	10	92

Fig. 8a shows the Nyquist plots of the NaCu_0.2_Fe_0.8−*x*_Mn_*x*_O_2_ samples (*x* = 0.4, 0.5, 0.6, 0.7) before grinding. All samples exhibit typical Nyquist plots consisting of two overlapping semicircles in the high-to-medium-frequency regions, followed by a sloped segment at low frequencies. These features correspond to contributions from the electrode/electrolyte interface, charge-transfer resistance, and sodium-ion diffusion, respectively. This shape is similar to that reported for sodium-layered oxides in the literature.^63^ The solution resistance (*R*_S_) values are relatively low (1–3 Ω) and similar for all samples because of the use of the same electrolyte for all the investigated electrodes. In addition, the solid-electrolyte interphase (SEI) layer contributes an interfacial resistance ranging ranging from 3.5 to 10 Ω, depending on the electrode composition. The major component of the total resistance is the charge-transfer resistance (*R*_CT_), which varies from 63 to 240 Ω. As expected, the lowest *R*_CT_ value is observed for the sample with *x* = 0.6, which aligns well with its superior electrochemical performance.

The relationship between *Z*′ and *ω*^−0.5^ is shown in Fig. S7, and the slope is defined as the Warburg factor (*σ*). Based on linear fit results, the sample with *x* = 0.7 has the smallest slope, which signifies the highest diffusion coefficient.^64^ The Na^+^ diffusion coefficient (*D*_Na^+^_) can be calculated according to the following equation:^65,66^
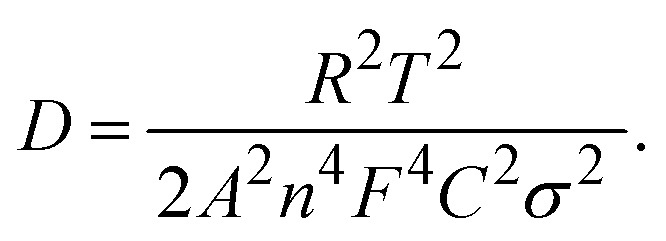


In this equation, *D* is the Na^+^ diffusion coefficient, *R* is the ideal gas constant, *T* is the absolute temperature, *A* is the area of the surface of the electrode, *n* is the number of electrons participating in the reaction, *F* is the Faraday constant, *C* is the molar concentration of Na^+^, and *σ* is the Warburg coefficient. Table S4 summarizes the obtained values of the Warburg coefficient and diffusion coefficient. These results clearly demonstrate that the Na^+^ diffusion coefficient increases significantly with increasing Mn content. In particular, the *x* = 0.7 sample exhibits the highest ionic diffusion coefficient, indicating enhanced sodium-ion transport kinetics, which contributes to its superior electrochemical performance.

Fig. 8b compares the EIS data for the NaCu_0.2_Fe_0.8−*x*_Mn_*x*_O_2_ sample with *x* = 0.7 before and after grinding, as this composite demonstrates the best electrochemical rate capability after grinding. The fitted parameters reveal a decrease in the charge-transfer resistance from 120 to 92 Ω, attributed to the reduced particle size, which facilitates sodium-ion diffusion in the electrode. Meanwhile, the SEI resistance (*R*_SEI_) increases slightly from 7.8 to 10 Ω due to the increased surface area resulting from the grinding process; this trend has been observed for layered oxide systems.^67^ The SEM of the NaCu_0.2_Fe_0.8−*x*_Mn_*x*_O_2_ sample with *x* = 0.7 before and after cycling was performed, and the micrographs are presented in Fig. S8. As observed in Fig. S8, the SEM images do not show significant morphological changes in the electrode before and after cycling. Before cycling (images a and b), the electrode surface is uniform, and the particles of the active material are well distributed. In addition, no visible cracks are detected, confirming the successful electrode preparation. After cycling (images c and d), the surface shows a slight increase in porosity due to electrochemical reactions and contact with the electrolyte; however, no microstructural damage or cracks are detected after cycling. This suggests structural stability of our prepared electrodes during cycling.

## 4 Conclusion

NaCu_0.2_Fe_0.8−*x*_Mn_*x*_O_2_ (*x* = 0.4, 0.5, 0.6, 0.7) materials were evaluated as promising cathode materials for advanced sodium ion batteries. These samples crystallized in the hexagonal system. The crystalline structures were identified with O3 and P3 types for samples with *x* = 0.4 and 0.5 and for samples with *x* = 0.6 and 0.7, respectively. Moreover, through Mössbauer spectroscopy, the valence state of iron in both samples proved to be high-spin Fe^3+^ located at two sites. Furthermore, the conducted electrochemical measurements demonstrated that as the Mn percentage increased, the specific capacity improved. Furthermore, cyclic voltammetry at different scan rates was equally performed for samples with *x* = 0.4 and 0.7. Assessing the dependency of the peak current and the square root (*v*^1/2^) of the scan rates indicated linear behavior, which confirmed the structural stability of the samples. The second part of this paper was devoted to studying the effect of particle size reduction on the electrochemical properties. The structural study revealed that the samples with *x* = 0.4 and 0.5 had the O3 structure type, whereas the samples with *x* = 0.6 and 0.7 changed after grinding to the O3 and P2 structure types, respectively. We confirmed that lower particle sizes generated the largest specific area and thereby enhanced electrochemical performance. Moreover, potentiostatic electrochemical impedance spectroscopy measurements showed the role of the electrode/electrolyte interface, charge-transfer resistance, and sodium-ion diffusion in performance. Finally, the SEM micrographs of NaCu_0.2_Fe_0.8−*x*_Mn_*x*_O_2_ (*x* = 0.7) did not show significant morphological changes in the electrode before and after cycling, which demonstrated the high stability of this cathode material during cycling.

## Author contributions

Ichrak Ben Slima: investigation, data curation, writing – original draft, writing – review and editing. Kawthar Trabelsi: investigation, data curation, writing – original draft. Lahcen Fkhar: investigation, data curation, writing – original draft, writing – review and editing. Karim Karoui: methodology, investigation. Frédéric Boschini: resources, project administration, funding acquisition. Abdallah Ben Rhaiem: writing – review and editing, writing – original draft, supervision, methodology, investigation, funding acquisition, conceptualization, visualization, validation. Abdelfattah Mahmoud: writing – review and editing, supervision, funding acquisition, methodology, investigation, conceptualization, validation, visualization.

## Conflicts of interest

The authors declare that they have no known competing financial interests or personal relationships that could have appeared to influence the work reported in this paper.

## Supplementary Material

RA-OLF-D6RA02392D-s001

## Data Availability

All data supporting the findings of this study are included within the manuscript. Supplementary information (SI) is available. See DOI: https://doi.org/10.1039/d6ra02392d.
