# Supplementary material for: Synergistic effect of Mn substitution and ball milling on NaCu0.2Fe0.8−xMnxO2 cathode materials for sodium-ion batteries
Source: RSC Adv. 2026 Jul 2;16(34):33027–39. doi: 10.1039/d6ra02392d (PMC13326591; doi:10.1039/d6ra02392d)
Supplement: RA-016-D6RA02392D-s001 [file RA-016-D6RA02392D-s001.pdf]

Supplementary Information (SI) for RSC Advances.
This journal is © The Royal Society of Chemistry 2026

Supplementary information

Synergistic effect of Mn Substitution and Ball Milling on $NaCu_{0.2}Fe_{0.8-x}Mn_xO_2$ Cathode materials for Sodium-Ion Batteries

Ichrak Ben Slima[a], Kawthar Trabelsi[a], Lahcen Fkhar[b], Karim Karoui[a,c], Frédéric Boschini[b], Abdallah Ben Rhaiem[a,*] Abdelfattah Mahmoud[b*]
[a] Laboratory LaSCOM, University of Sfax, BP1171, 3000, Sfax, Tunisia
[b] GREENMAT, CESAM, Institute of Chemistry B6, University of Liège, 4000 Liège, Belgium
[c] GREMAN UMR 7347-CNRS, IUT of Blois, University of Tours, Blois, France

***Corresponding author:**
E-mail address: **abdallahrhaiem@yahoo.fr/abdelfattah.mahmoud@uliege.be.**

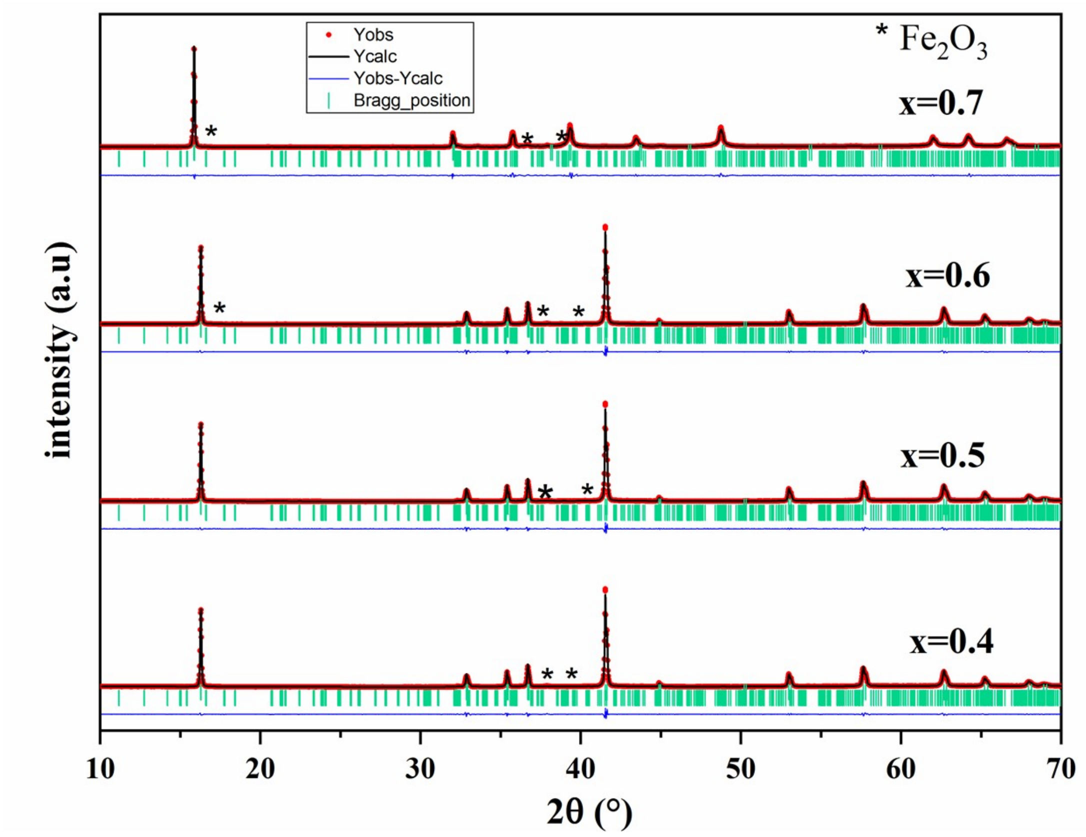

Figure S1 : XRD pattern associated to $NaCu_{0.2}Fe_{0.8-x}Mn_xO_2$ samples (x= 0.4; 0.5; 0.6; 0.7)

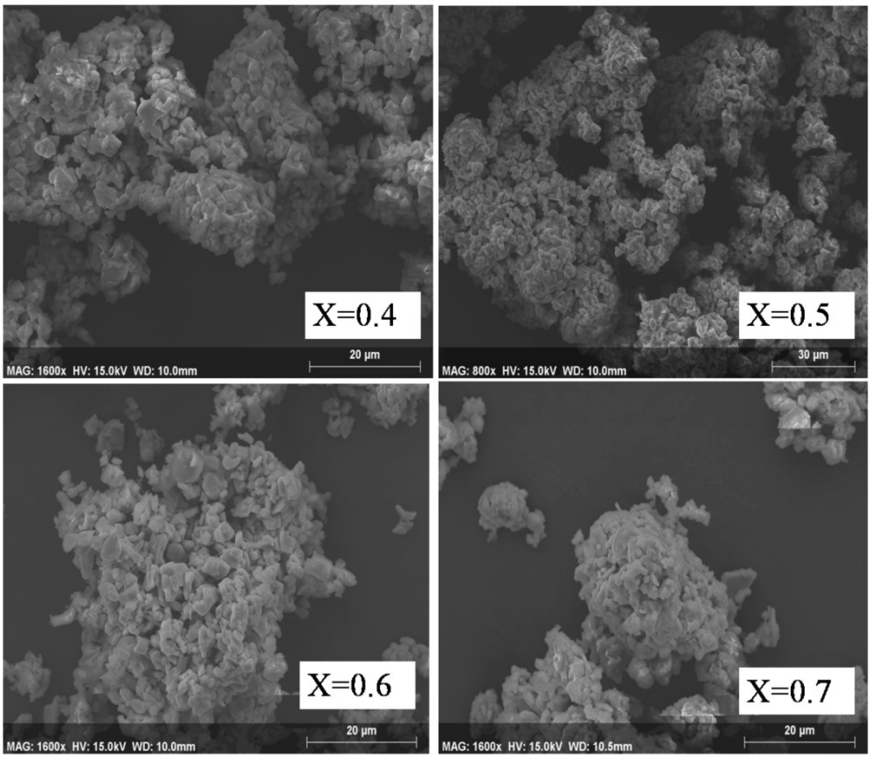

Figure S2: SEM micrographs of $NaCu_{0.2}Fe_{0.8-x}Mn_xO_2$ samples (x= 0.4; 0.5; 0.6; 0.7).

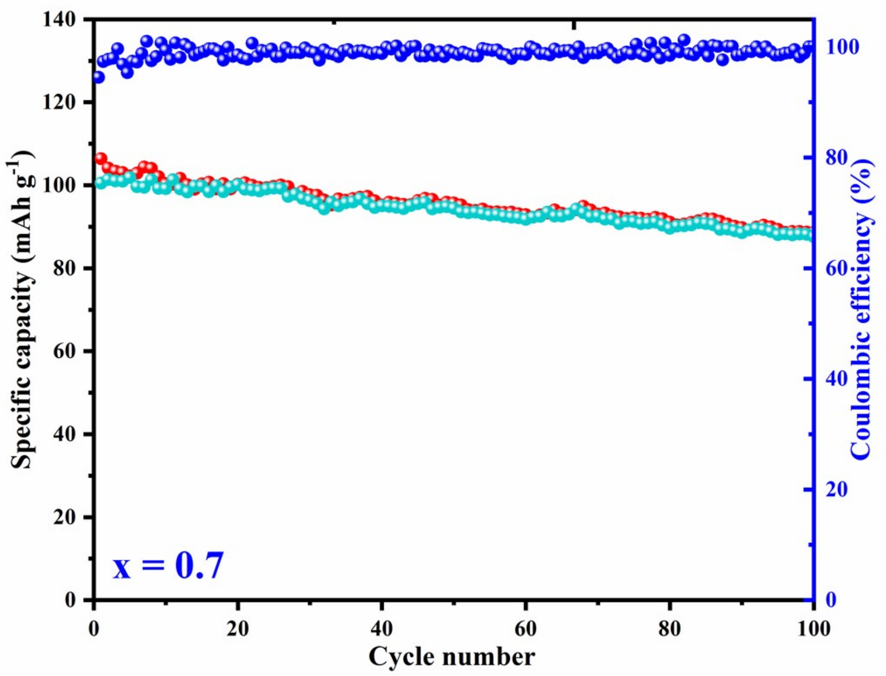

Figure S3: Cycling performance of $NaCu_{0.2}Fe_{0.8-x}Mn_xO_2$ (x= 0.7) sample at C/20 rate.

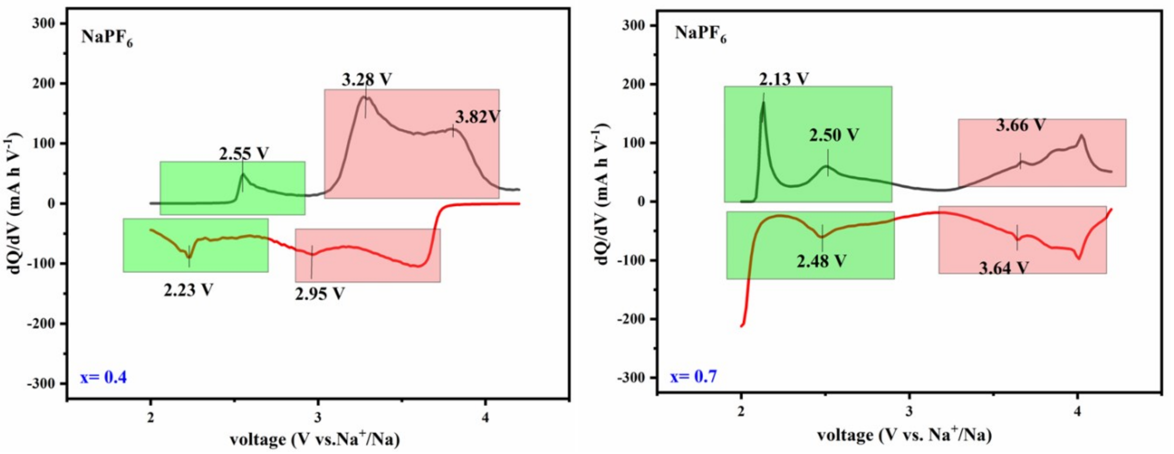

Figure S4: The dQ/dV plot of the $NaCu_{0.2}Fe_{0.8-x}Mn_xO_2$ (x = 0.4; 0.7) cathode materials for sodium-ion batteries at the first cycle.

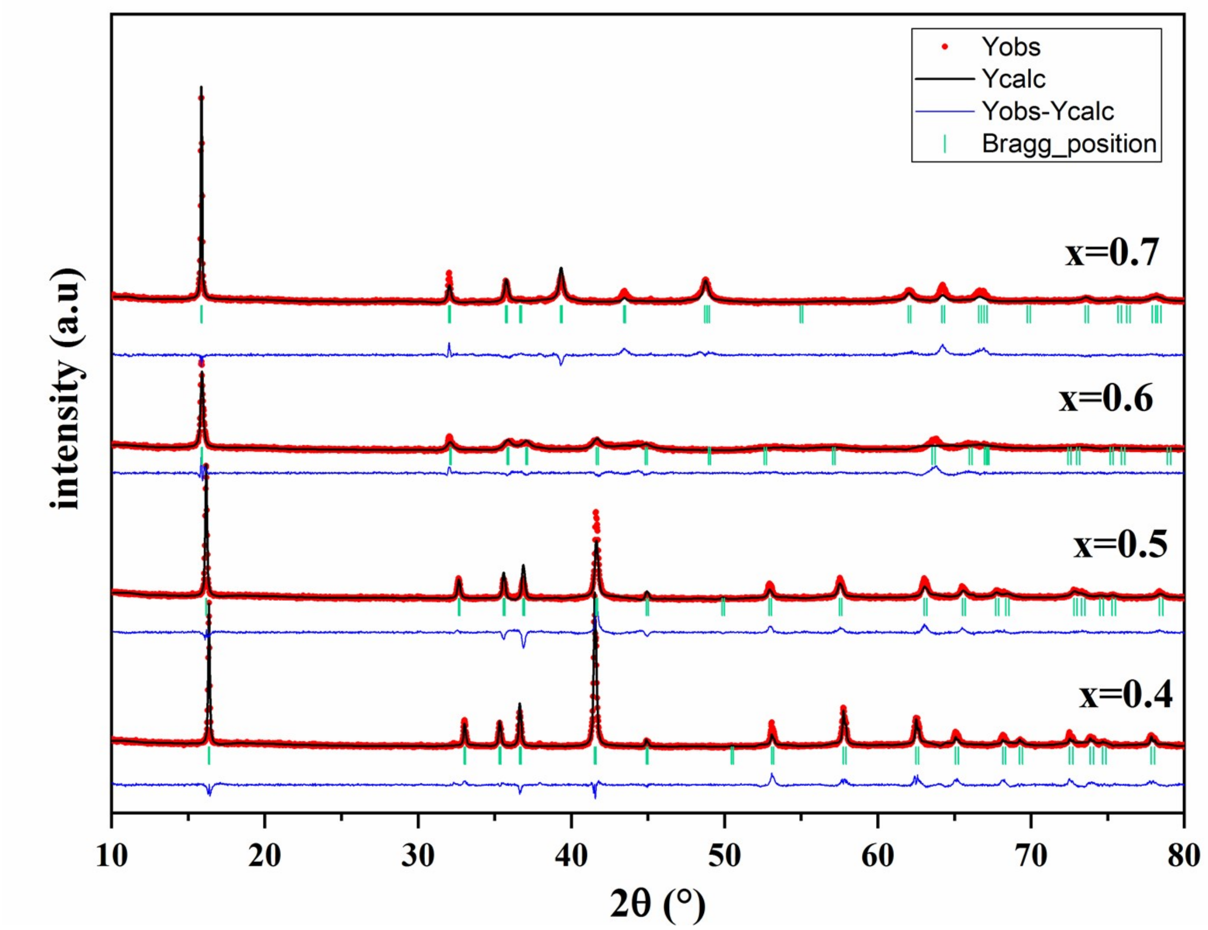

Figure S5: XRD pattern associated to ground $NaCu_{0.2}Fe_{0.8-x}Mn_xO_2$ samples (x= 0.4; 0.5; 0.6; 0.7)

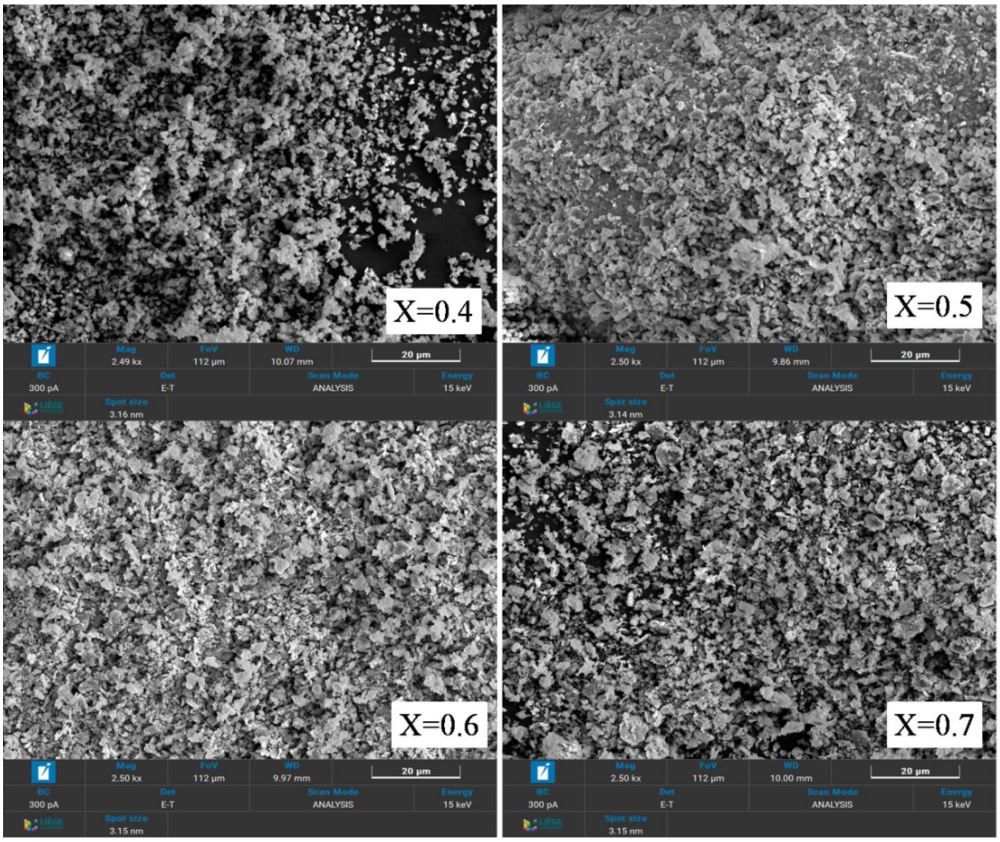

Figure S6: SEM micrographs of ground $NaCu_{0.2}Fe_{0.8-x}Mn_xO_2$ samples (x= 0.4; 0.5; 0.6; 0.7)

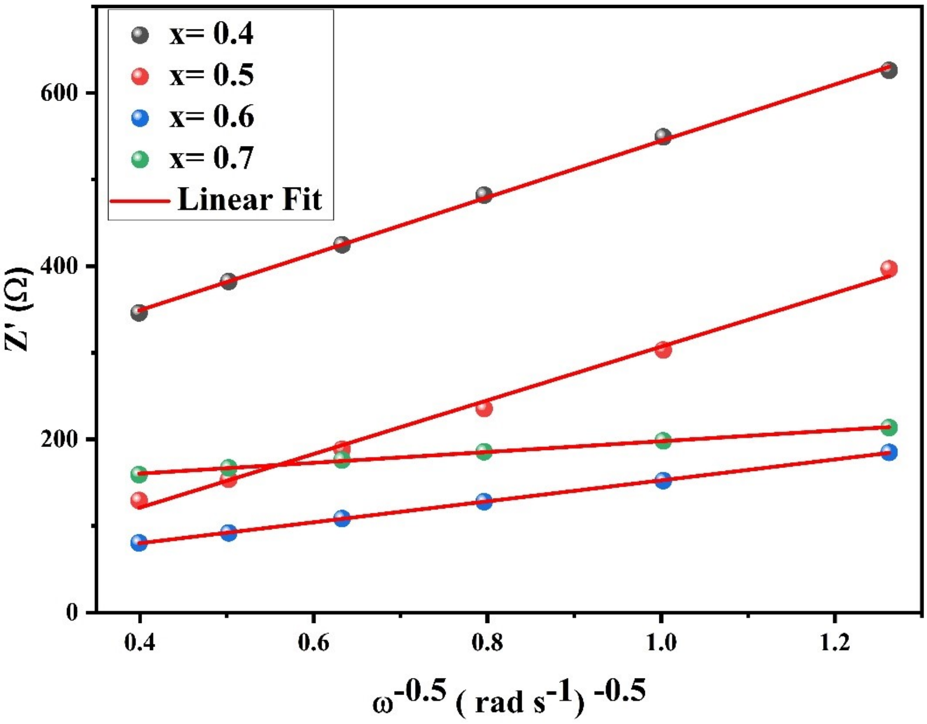

Figure S7: the relationship between Z′ against $\omega^{0.5}$ of the $NaCu_{0.2}Fe_{0.8-x}Mn_xO_2$(x = 0.4; 0.5; 0.6; 0.7) samples.

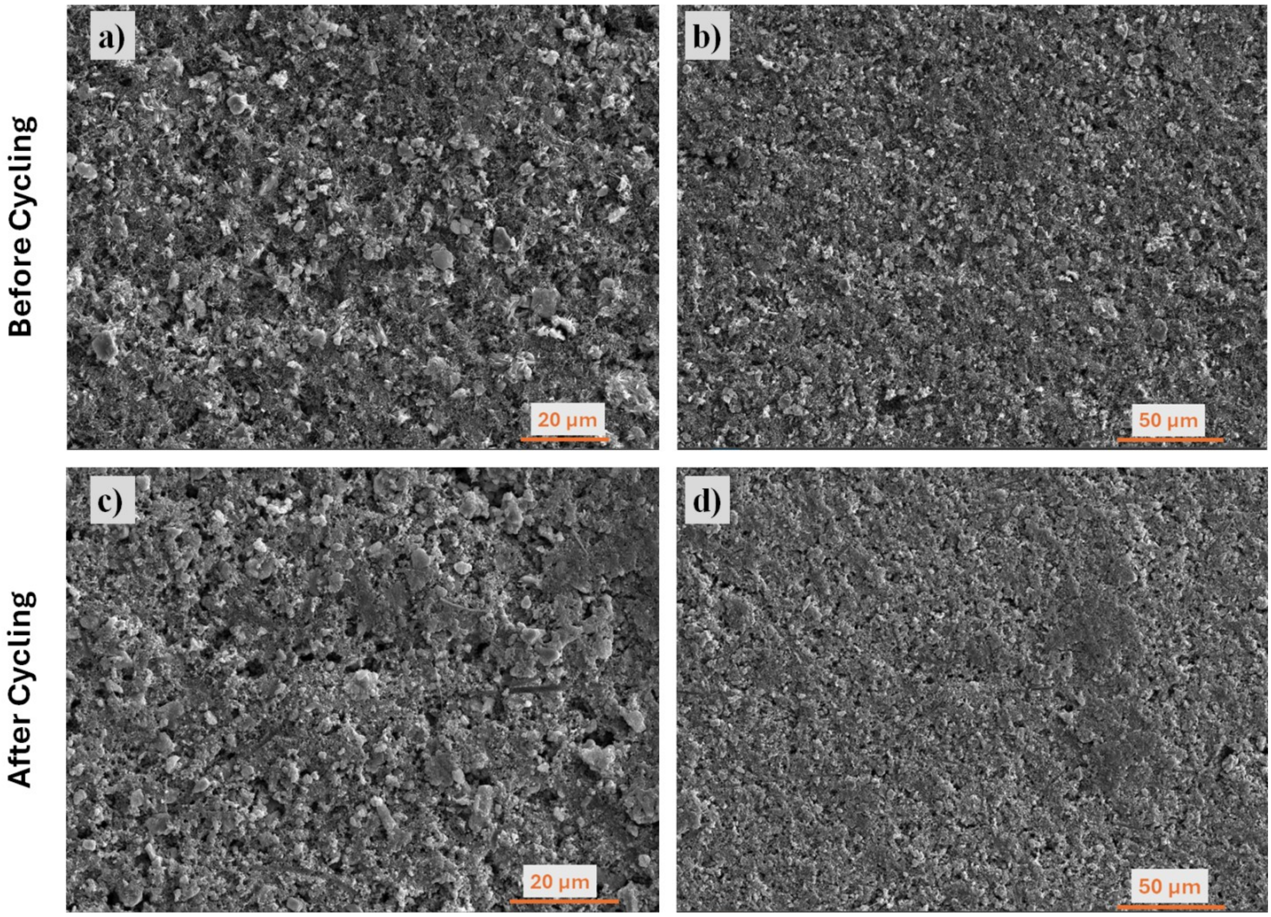

Figure S8: SEM micrographs of $NaCu_{0.2}Fe_{0.8-x}Mn_xO_2$ (x=0.7) Electrodes (a, b) Before cycling, (c, d) after cycling

Table S1 : Refined cell parameters of the $NaCu_{0.2}Fe_{0.8-x}Mn_xO_2$(x = 0.4; 0.5; 0.6; 0.7) samples.

X	Space group	a(Å)	c(Å)	χ^2
**0.4**	R-3m	2.962(1)	16.341(7)	3.77
**0.5**	R-3m	2.938(1)	16.520(11)	2.42
**0.6**	R-3m	2.810(2)	16.816(5)	5.28
**0.7**	R-3m	2.732(5)	16.760(4)	5.51

Table S.2: Cell parameters obtained by the Rietveld refinement of ground $NaCu_{0.2}Fe_{0.8-x}Mn_xO_2$(x = 0.4; 0.5; 0.6; 0.7) samples**.**

X	Space group	a(Å)	c(Å)	χ^2
**0.4**	R-3m	2.970(1)	16.2689(13)	3.60
**0.5**	R-3m	2.947(2)	16.453(3)	4.52
**0.6**	R-3m	2.925(7)	16.734(9)	2.30
**0.7**	$P6_3/mmc$	2.900(2)	11.175(2)	3.69

Table S.3: Atomic positional parameters and structure type of ground $NaCu_{0.2}Fe_{0.8-x}Mn_xO_2$ samples (x= 0.4; 0.5; 0.6; 0.7)

Table S.3.a: X=0.4 / O3 type

**Atom**	**Wyck.**	**x**	**y**	**z**	**U**
**Na**	3b	0	0	0.5	0.064(3)
**Cu**	3a	0	0	0	0.0318
**Fe**	3a	0	0	0	0.0318
**Mn**	3a	0	0	0	0.0318
**O**	6c	0	0	0.2585(4)	0.0259

Table S.3.b: X=0.5 / O3 type

**Atom**	**Wyck.**	**x**	**y**	**z**	**U**
**Na**	3b	0	0	0.5	0
**Cu**	3a	0	0	0	0
**Fe**	3a	0	0	0	0
**Mn**	3a	0	0	0	0
**O**	6c	0	0	0.262(1)	0

Table S.3.c: X=0.6 / O3 type

**Atom**	**Wyck.**	**x**	**y**	**z**	**U**
**Na**	3b	0	0	0.5	0.2054
**Cu**	3a	0	0	0	0.1985
**Fe**	3a	0	0	0	0.1985
**Mn**	3a	0	0	0	0.1985
**O**	6c	0	0	0.21765	0.0987

Table S.3.d: X=0.7 / P2 type

**Atom**	**Wyck.**	**x**	**y**	**z**	**U**
**Na1**	2b	0	0	1/4	0
**Na2**	2d	2/3	1/3	1/4	0
**Cu**	2a	0	0	0	0
**Fe**	2a	0	0	0	0
**Mn**	2a	0	0	0	0
**O**	4f	1/3	2/3	0.0785(1)	0

Table S4: Warburg coefficient and Diffusion coefficient of the $NaCu_{0.2}Fe_{0.8-x}Mn_xO_2$ (x = 0.4; 0.5; 0.6; 0.7) samples.

Sample	σ (Ω (rad $s^{-1})^{-0.5}$)	$D_{(Na^+)}$ ($10^{-15} cm^2 s^{-1}$)
x=0.4	325	3
x=0.5	309	3.5
x=0.6	120	20
x=0.7	62	70
